# Temporal changes in concentrations of lipids and apolipoprotein B among adults with diagnosed and undiagnosed diabetes, prediabetes, and normoglycemia: findings from the National Health and Nutrition Examination Survey 1988–1991 to 2005–2008

**DOI:** 10.1186/1475-2840-12-26

**Published:** 2013-01-30

**Authors:** Earl S Ford, Chaoyang Li, Allan Sniderman

**Affiliations:** 1 Division of Population Health, National Center for Chronic Disease Prevention and Health Promotion, Centers for Disease Control and Prevention, Atlanta, GA, USA; 2 Division of Behavioral Surveillance, Office of Surveillance, Epidemiology, and Laboratory Services, Centers for Disease Control and Prevention, Atlanta, GA, USA; 3 Mike Rosenbloom Laboratory for Cardiovascular Research, McGill University Health Centre, Montreal, Quebec, Canada

**Keywords:** Apolipoprotein B, Cholesterol, Diabetes, High-density lipoprotein cholesterol, Lipids, Prediabetes, Triglycerides, Fenofibrate, Gemfibrozil, Niacin

## Abstract

**Background:**

Diabetes is characterized by profound lipid abnormalities. The objective of this study was to examine changes in concentrations of lipids and apolipoprotein B among participants stratified by glycemic status (diabetes, undiagnosed diabetes, prediabetes, and normoglycemia) in the United States from 1988–1991 to 2005–2008.

**Methods:**

We used data from 3202 participants aged ≥20 years from the National Health and Nutrition Examination Survey (NHANES) III (1988–1991) and 3949 participants aged ≥20 years from NHANES 2005–2008.

**Results:**

Among participants of all four groups, unadjusted and adjusted mean concentrations of total cholesterol, low-density lipoprotein cholesterol, non-high-density lipoprotein cholesterol, and apolipoprotein B, but not triglycerides, decreased significantly. Among participants with prediabetes and normoglycemia, unadjusted and adjusted mean concentrations of high-density lipoprotein cholesterol increased significantly. Adjusted mean log-transformed concentrations of triglycerides decreased in adults with undiagnosed diabetes and prediabetes. During 2005–2008, unadjusted concentrations of apolipoprotein B ≥80 mg/dl were observed in 72.8% of participants with diagnosed diabetes, 87.9% of participants with undiagnosed diabetes, 86.6% of participants with prediabetes, and 77.2% of participants with normoglycemia. The unadjusted use of cholesterol-lowering medications rose rapidly, especially among participants with diabetes (from ~1% to ~49%, P <0.001). The use of fenofibrate, gemfibrozil, and niacin rose significantly only among adults with diagnosed diabetes (from ~2% to ~8%, P = 0.011).

**Conclusion:**

Lipid profiles of adults with diabetes improved during the approximately 16-year study period. Nevertheless, large percentages of adults continue to have elevated concentrations of apolipoprotein B.

## Background

During the last several decades, diabetes has emerged as a major public health problem in the United States as the incidence and prevalence of obesity has escalated rapidly [1,2]. The lifetime probability of developing this disease is approximately 32.8% for men and 38.5% for women [3], and an estimated 25.6 million adults have diabetes [4]. Furthermore, diabetes has an enormous impact on health care costs in the United States; in 2007, for example, the economic costs attributable to this condition were estimated to have been $174 billion [4].

Much of the toll exacted by diabetes is due to the complications of this disease, notably from cardiovascular disease: as many as 65% of people with diabetes will die of cardiovascular disease, and diabetes approximately doubles the risk of developing cardiovascular disease [5,6]. This increased risk for cardiovascular morbidity and mortality is largely a function of a number of cardiometabolic abnormalities, including lipid abnormalities. In general, people with diabetes tend to have elevated concentrations of triglycerides and apolipoprotein B, low concentrations of high-density lipoprotein cholesterol, and an elevated number of small dense low-density lipoprotein cholesterol particles [7].

Previous trend studies have shown some improvement in lipid abnormalities in adults with diabetes including total cholesterol, low-density lipoprotein cholesterol, and non-high-density lipoprotein cholesterol [8-10]. However, less is known about apolipoprotein B, which some have argued is a superior marker of cardiovascular risk than low-density lipoprotein cholesterol or non-high-density lipoprotein cholesterol [11]. Consequently, the objective of the present study was to examine changes in concentrations of lipids with a focus on apolipoprotein B among adults with diagnosed and undiagnosed diabetes from 1988–1991 to 2005–2008. In addition, we examined changes in concentrations of lipids and apolipoprotein B among participants with prediabetes and normoglycemia.

## Research design and methods

The analyses for the present study were conducted using data from the first phase of the National Health and Nutrition Examination Survey (NHANES) III (1988–1991) and from NHANES 2005–2008. Using a multistage, stratified sampling design, participants were recruited into the survey. After an interview in the home, participants were invited to attend one of three examination sessions: morning, afternoon, or evening. Persons attending the morning session were asked to fast overnight. In the mobile examination center, participants completed additional questionnaires, had a series of examinations, and provided biological specimens including blood. Interview and examination response rates were 86% and 78%, respectively, for NHANES III, 80% and 77%, respectively, for NHANES 2005–2006, and 78% and 75%, respectively, for NHANES 2007–2008. Details about the surveys and their methods have been published previously [12,13].

Four groups of participants were defined: those with diagnosed diabetes, those with undiagnosed diabetes, those with prediabetes, and those with normoglycemia. Participants with diagnosed diabetes were identified by responses to the NHANES questions: “Have you ever been told by a doctor that you have diabetes or sugar diabetes?” or “Have you ever been told by a doctor or health professional that you have diabetes or sugar diabetes?”, respectively, in the two surveys. Those who responded “yes” were considered to have diagnosed diabetes. Participants who responded that they may have had "borderline" diabetes or were "prediabetic" were coded as not having diagnosed diabetes. Concentrations of HbA1c were used to establish the glycemic status of the remaining participants without diagnosed diabetes as follows: undiagnosed diabetes: ≥6.5%; prediabetes: 5.7- < 6.5%; and normal: <5.7%.

High-performance liquid chromatography (HPLC) was used to measure concentrations of HbA1c in NHANES III (Bio-Rad DIAMAT glycosylated hemoglobin analyzer system, Bio-Rad Laboratories, Hercules, CA) and NHANES 2005–2008 (NHANES 2005–2006: Tosoh A1c 2.2 Plus Glycohemoglobin Analyzer, Tosoh Medics, Inc., So. San Francisco, CA; NHANES 2007–2008: Tosoh G 7 HPLC Glycohemoglobin Analyzer, Tosoh Medics, Inc., So. San Francisco, CA). Serum concentrations of total cholesterol, high-density lipoprotein cholesterol, and triglycerides were measured enzymatically on a Hitachi 704 Analyzer (Boehringer Mannheim Diagnostics, Indianapolis, IN) for NHANES III, on a Hitachi 717 or 912 Analyzer (Roche Diagnostics, Indianapolis, IN) for NHANES 2005–2006, and on a Roche Modular P Chemistry Analyzer (Roche Diagnostics, Indianapolis, IN) for NHANES 2007–2008. Measurements of apolipoprotein B for NHANES III were performed by using radial immunodiffusion (Behring Diagnostics, Westwood, MA) and rate immunonephelometrix assay (Beckman Instruments, Brea, CA). Nephelometry was used to measure apolipoprotein B during 2005–2008 (NHANES 2005–2006: Dade Behring BN100 nephelometer, Dade Behring, Deerfield, IL; NHANES 2007–2008: ProSpec nephelometer, Dade Behring GMBH, Marburg, Germany).

Participants could report up to 16 medications in NHANES III and 20 medications in NHANES 2005–2008. When prescription bottles were available, participants were asked to show them to the interviewers. For our study, we established the following two groups of medications: cholesterol-lowering medications (statins [atorvastatin, fluvastatin, lovastatin, pravastatin, rosuvastatin, simvastatin], ezetimibe, cholestyramine, probucol, colesevelam, and colestipol) and triglyceride-lowering medications (fenofibrate, gemfibrozil, and niacin). Products containing a cholesterol-lowering medication in the form of a statin and a triglyceride-lowering medication were counted as one of each.

The following covariates were included in the analyses: age, gender, race or ethnicity (whites, African Americans, Mexican Americans, other), educational status (<high school, high school graduate or equivalent, >high school), and body mass index. Measured weight and height were used to calculate and categorize body mass index levels (<25, 25- < 30, and ≥30 kg/m^2^).

For participants with diagnosed diabetes, we also calculated 10-year predicted cardiovascular risk using the UK Prospective Diabetes Study (UKPDS) risk engine that uses the following variables: age at diagnosis of diabetes, gender, race or ethnicity, smoking status, concentration of HbA1c, systolic blood pressure, and ratio of concentrations of total cholesterol to high-density lipoprotein cholesterol [14]. Participants who had smoked at least 100 cigarettes during their lifetime and were currently smoking were designated as current smokers. For systolic blood pressure, we averaged the second and third measurements of systolic blood pressure.

The analyses were limited to participants aged ≥20 years who attended the morning examination and had fasted ≥8 hours. Pregnant women were excluded. Adjusted mean concentrations of lipids and apolipoprotein B were calculated by using analysis of covariance. Differences in percentages and means were tested by using chi-square tests and t-tests, respectively. In addition, changes in the prevalence of abnormal concentrations of lipids were examined by log-linear regression analysis adjusting for age (continuous) and age, gender, race or ethnicity, educational status, and body mass index (continuous). Data management was done with SAS and estimates were produced with SUDAAN to account for the complex sampling designs of the surveys. Sample sizes reflect unweighted numbers, whereas sampling weights were used to generate estimates.

## Results

Of the 3840 participants aged ≥20 years in NHANES III who attended the morning examination, 3510 were left after excluding pregnant women and participants who had fasted <8 hours. Additional exclusions of participants who had missing data for variables needed to establish glycemic status and for lipids reduced the sample size to 3225 participants. After covariates with missing values were excluded, 3202 were included in the analyses. In NHANES 2005–2008, 4341 participants attended the morning examination, 4173 remained after excluding pregnant women and participants who had fasted <8 hours, 4020 were left after excluding participants who had missing data for variables needed to establish glycemic status and for lipids, and 3949 were included in the analyses after excluding participants who had covariates with missing values. Changes in covariates by glycemic status are shown in Table 1.

**Table 1:** Unadjusted percentages, medians, or means (standard error) of selected sociodemographic and anthropometric characteristics among adults aged > =20 years, National Health and Nutrition Examination Survey 1988–1991 and 2005-2008

	**Diagnosed diabetes**			**Undiagnosed diabetes**			**Prediabetes**			**Normal**		
	**1988-1991**	**2005-2008**	P*	**1988-1991**	**2005-2008**	P*	**1988-1991**	**2005-2008**	P*	**1988-1991**	**2005-2008**	P*
N	207	457		92	103		566	850		2337	2539	
Age (years) - median	59.9 (1.6)	59.8 (1.1)	—	58.6 (2.1)	61.6 (3.0)	—	56.9 (2.2)	56.5 (0.9)	—	38.0 (0.8)	40.9 (0.8)	—
Age (%)			0.783			0.531			0.257			0.001
20-39 years	13.3 (4.3)	10.8 (1.7)		13.2 (4.8)	6.7 (3.0)		18.0 (2.7)	14.7 (1.4)		51.5 (1.6)	46.2 (1.4)	
40-59 years	33.6 (4.8)	37.3 (2.6)		38.1 (6.5)	40.8 (6.2)		36.2 (3.3)	41.2 (1.9)		30.6 (1.5)	38.8 (1.4)	
≥60 years	53.1 (3.8)	51.9 (2.9)		48.7 (6.6)	52.5 (6.5)		45.9 (4.3)	44.1 (2.1)		17.9 (1.5)	15.0 (1.4)	
Men (%)	36.4 (5.7)	44.4 (3.0)	0.221	49.5 (7.9)	56.6 (6.1)	0.482	53.3 (3.1)	47.9 (2.3)	0.170	47.9 (1.3)	48.8 (1.0)	0.567
Race or ethnicity (%)			0.268			0.932			0.020			0.004
White	73.4 (4.3)	65.3 (4.3)		53.3 (6.3)	55.3 (6.9)		63.9 (5.0)	66.7 (3.4)		81.1 (2.2)	73.7 (2.0)	
African American	15.1 (3.6)	17.3 (2.9)		29.8 (4.9)	28.3 (4.0)		24.0 (3.1)	17.4 (2.5)		7.7 (1.0)	8.3 (1.1)	
Mexican American	8.4 (1.4)	8.2 (1.4)		8.1 (2.6)	9.9 (2.7)		4.8 (0.5)	7.3 (1.3)		4.7 (0.6)	8.0 (0.9)	
Other	3.1 (2.2)	9.2 (2.3)		8.8 (5.9)	6.6 (3.1)		7.3 (2.4)	8.6 (1.9)		6.5 (1.7)	9.9 (1.1)	
≥High school graduates (%)	57.4 (5.5)	70.4 (2.2)	0.030	50.2 (5.4)	71.7 (4.8)	0.013	60.3 (2.8)	77.7 (1.7)	<0.001	79.4 (1.9)	84.4 (1.2)	0.034
Body mass index ≥30 kg/m^2^ (%)	38.2 (5.8)	61.7 (2.8)	0.001	60.3 (7.1)	68.4 (6.2)	0.378	28.2 (2.5)	43.0 (2.0)	<0.001	17.4 (1.3)	27.5 (1.3)	<0.001
Body mass index (kg/m^2^)	29.6 (0.7)	32.8 (0.5)	0.001	32.6 (1.8)	34.8 (1.3)	0.343	27.6 (0.3)	30.2 (0.3)	<0.001	25.7 (0.2)	27.5 (0.2)	<0.001
HbA1c (%)	6.8 (0.2)	7.2 (0.1)	0.060	7.8 (0.3)	7.5 (0.2)	0.345	5.9 (<0.1)	5.9 (<0.1)	0.024	5.0 (<0.1)	5.2 (<0.1)	<0.001

*P-value from chi-square test for categorical variables and from t-test for means.

Among participants with diagnosed diabetes, undiagnosed diabetes, prediabetes, and normoglycemia, significant decreases occurred in concentrations of total cholesterol, low-density lipoprotein cholesterol, non-high-density lipoprotein cholesterol, and apolipoprotein B (Table 2). Concentrations of high-density lipoprotein cholesterol also increased significantly among participants with prediabetes and normoglycemia in all models and among participants with diagnosed diabetes in the most adjusted model. Log-transformed concentrations of triglycerides decreased significantly among participants with undiagnosed diabetes and prediabetes only in the most adjusted model.

**Table 2:** Unadjusted and least-square adjusted mean concentrations (standard error) of lipids and apolipoprotein B among adults aged ≥20 years, by glycemic status and survey, National Health and Nutrition Examination Surveys 1988–1991 and 2005-2006

	**Diagnosed diabetes**			**Undiagnosed diabetes**			**Prediabetes**			**Normal**		
	**1988-1991**	**2005-2008**	P*	**1988-1991**	**2005-2008**	P*	**1988-1991**	**2005-2008**	P*	**1988-1991**	**2005-2008**	P*
N	207	457		92	103		566	850		2337	2539	
**Unadjusted**												
Total cholesterol (mg/dl)	215.0 (3.6)	181.0 (2.3)	<0.001	223.0 (5.0)	200.4 (4.8)	0.002	218.0 (1.7)	202.9 (1.8)	<0.001	200.5 (1.4)	195.0 (0.9)	0.002
High-density lipoprotein cholesterol (mg/dl)	49.1 (1.6)	50.7 (1.0)	0.390	44.9 (1.8)	47.7 (1.6)	0.234	48.0 (0.7)	53.6 (0.7)	<0.001	51.8 (0.7)	55.7 (0.4)	<0.001
Low-density lipoprotein cholesterol (mg/dl)	135.4 (2.9)	100.5 (1.8)	<0.001	143.3 (3.8)	121.7 (4.3)	<0.001	142.6 (1.8)	122.3 (1.4)	<0.001	124.8 (1.3)	115.4 (0.9)	<0.001
Non-high-density lipoprotein cholesterol (mg/dl)	166.0 (4.4)	130.3 (2.0)	<0.001	178.1 (4.6)	152.7 (4.4)	<0.001	170.0 (1.8)	149.3 (1.8)	<0.001	148.8 (1.5)	139.3 (1.0)	<0.001
Triglycerides (mg/dl)†	153.1 (9.8)	148.9 (4.0)	0.687	173.6 (10.1)	155.4 (10.1)	0.207	137.0 (4.2)	136.9 (3.6)	0.985	119.6 (3.6)	119.5 (1.7)	0.974
Triglycerides (mg/dl)‡	137.8 (9.7)	133.0 (3.6)	0.634	158.4 (9.2)	135.0 (9.4)	0.084	122.0 (3.5)	122.1 (2.9)	0.970	104.3 (3.2)	104.6 (1.6)	0.933
Apolipoprotein B (mg/dl)	114.2 (2.7)	96.3 (1.5)	<0.001	120.3 (3.4)	110.4 (3.1)	0.035	115.6 (1.3)	106.2 (1.2)	<0.001	102.0 (0.9)	98.6 (0.8)	0.011
**Age-adjusted**												
Total cholesterol (mg/dl)	206.7 (3.5)	172.3 (2.5)	<0.001	215.2 (4.5)	191.3 (4.7)	0.001	211.0 (1.7)	195.9 (1.8)	<0.001	202.9 (1.4)	197.0 (0.8)	<0.001
High-density lipoprotein cholesterol (mg/dl)	47.5 (1.6)	49.1 (1.0)	0.418	43.4 (1.7)	46.0 (1.6)	0.265	46.7 (0.7)	52.3 (0.7)	<0.001	52.2 (0.7)	56.0 (0.4)	<0.001
Low-density lipoprotein cholesterol (mg/dl)	130.2 (2.8)	95.1 (2.0)	<0.001	138.5 (3.7)	116.0 (4.3)	<0.001	138.3 (1.8)	118.0 (1.5)	<0.001	126.3 (1.3)	116.7 (0.8)	<0.001
Non-high-density lipoprotein cholesterol (mg/dl)	159.2 (4.3)	123.3 (2.2)	<0.001	171.8 (4.3)	145.2 (4.3)	<0.001	164.3 (1.9)	143.7 (1.8)	<0.001	150.7 (1.5)	141.0 (0.9)	<0.001
Triglycerides (mg/dl)†	145.1 (9.6)	140.5 (4.2)	0.662	166.1 (9.8)	146.6 (9.7)	0.160	130.2 (4.4)	130.1 (3.5)	0.993	121.9 (3.6)	121.5 (1.6)	0.912
Triglycerides (mg/dl)‡	127.9 (8.9)	123.0 (3.6)	0.601	147.7 (8.3)	124.3 (8.3)	0.052	114.5 (3.3)	114.7 (2.5)	0.957	106.5 (3.2)	106.5 (1.5)	1.000
Apolipoprotein B (mg/dl)	109.9 (2.7)	91.9 (1.6)	<0.001	116.3 (3.2)	105.7 (3.1)	0.024	112.0 (1.3)	102.6 (1.2)	<0.001	103.2 (0.9)	99.7 (0.7)	0.006
**Adjusted for age, gender, race or ethnicity, education, and body mass index**												
Total cholesterol (mg/dl)	205.6 (3.3)	169.6 (2.5)	<0.001	213.0 (4.7)	187.6 (4.3)	<0.001	211.5 (1.8)	194.7 (1.9)	<0.001	203.7 (1.3)	197.0 (0.8)	<0.001
High-density lipoprotein cholesterol (mg/dl)	48.1 (1.2)	52.2 (1.0)	0.010	46.6 (2.0)	51.1 (2.1)	0.128	46.9 (0.7)	53.7 (0.7)	<0.001	51.2 (0.6)	56.0 (0.3)	<0.001
Low-density lipoprotein cholesterol (mg/dl)	129.2 (2.6)	91.8 (1.9)	<0.001	135.1 (4.2)	110.6 (3.8)	<0.001	138.0 (1.9)	116.3 (1.5)	<0.001	127.4 (1.3)	116.8 (0.7)	<0.001
Non-high-density lipoprotein cholesterol (mg/dl)	157.5 (3.9)	117.4 (2.2)	<0.001	166.4 (5.3)	136.4 (3.7)	<0.001	164.6 (1.9)	141.0 (1.9)	<0.001	152.6 (1.3)	141.0 (0.8)	<0.001
Triglycerides (mg/dl)†	141.7 (8.2)	128.2 (4.3)	0.153	156.3 (11.5)	129.6 (11.4)	0.100	133.0 (3.8)	125.2 (3.5)	0.137	125.9 (3.2)	120.9 (1.6)	0.161
Triglycerides (mg/dl)‡	124.4 (7.2)	111.0 (3.3)	0.078	136.3 (10.0)	108.0 (8.6)	0.032	117.5 (2.7)	110.1 (2.3)	0.041	110.2 (2.9)	105.9 (1.4)	0.179
Apolipoprotein B (mg/dl)	108.7 (2.5)	88.0 (1.5)	<0.001	112.5 (3.4)	99.7 (2.8)	0.005	111.8 (1.5)	100.8 (1.2)	<0.001	104.4 (0.9)	99.8 (0.6)	<0.001

*P-values from linear regression analyses.

†Untransformed mean.

‡Geometric mean.

A secondary observation of the results shown in Table 2 was that participants with undiagnosed diabetes and prediabetes exhibited worse lipid profiles than participants with diagnosed diabetes. During 2005–2008, unadjusted and adjusted concentrations of total cholesterol, low-density lipoprotein cholesterol, non-high-density lipoprotein cholesterol, and apolipoprotein B were significantly higher among participants with undiagnosed diabetes and prediabetes than among participants with diagnosed diabetes.

To estimate the possible impact of improvements in concentrations of total cholesterol and high-density lipoprotein cholesterol may have had on 10-year predicted risk from cardiovascular disease, we estimated risk using the UKPDS risk engine for the adults with diagnosed diabetes during 1988–1994 under two scenarios: 1) using the original data and 2) after reducing the ratio of TC/HDLC by approximately 20% for each participant while holding the values for the other variables that are part of the risk engine constant. The 20% reduction represents the mean improvement in the mean ratio of TC/HDLC from 1988–1994 to 2005–2008. The predicted 10-year risk dropped from 21.2% (95% confidence interval: 15.8—26.5) to 17.3% (95% confidence interval: 12.7—21.9).

**Figure 1:**
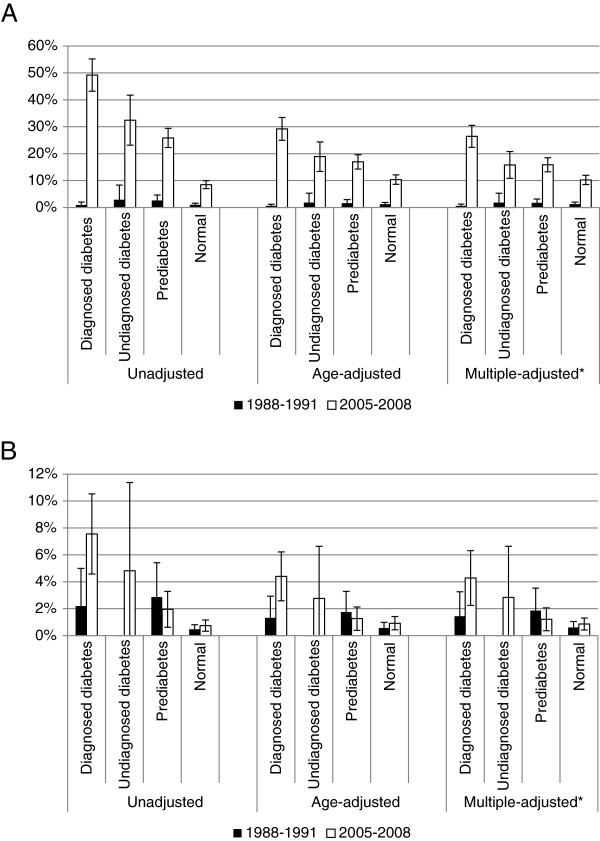
**Percentages (95% confidence interval) of participants aged ≥20 years who reported using cholesterol-lowering medications (panel A) and fenofibrate, gemfibrozil, or niacin (panel B), by survey period and glycemic status, National Health and Nutrition Examination Survey 1988–1991 and 2005–2006.** *Results were adjusted for age, gender, race or ethnicity, educational status, and body mass index.

The unadjusted use of cholesterol-lowering medications increased significantly in all groups (P for all comparisons <0.001) and was particularly striking among participants with diagnosed diabetes in whom the use increased from approximately 1% to 49% (Figure 1). The use of medications for lowering concentrations of triglycerides or increasing concentrations of high-density lipoprotein cholesterol also rose but at a less impressive rate. The largest increases were noticed in participants with diagnosed diabetes among whom the unadjusted percentage increased from about 2% to almost 8% (P = 0.011) and in participants with undiagnosed diabetes among whom the percentage increased from 0% to almost 5% (P = 0.147).

The analyses that generated the results shown in Table 2 were repeated after excluding participants who were using cholesterol-lowering medications, fenofibrate, gemfibrozil, and niacin (Table 3). Many of the significant improvements in lipid concentration persisted in analyses that excluded participants who used cholesterol-lowering and triglyceride-lowering medications, although the magnitude of the decrease narrowed to some degree in many comparisons.

**Table 3:** Unadjusted and least-square adjusted mean concentrations (standard error) of lipids and apolipoprotein B among adults aged > =20 years, by glycemic status and survey, National Health and Nutrition Examination Survey 1988–1991 and 2005–2006

	**Diagnosed diabetes**			**Undiagnosed diabetes**			**Prediabetes**			**Normal**		
	**1988-1991**	**2005-2008**	P*	**1988-1991**	**2005-2008**	P*	**1988-1991**	**2005-2008**	P*	**1988-1991**	**2005-2008**	P*
N	200	211		91	67		545	634		2302	2290	
**Unadjusted**												
Total cholesterol (mg/dl)	214.4 (3.7)	194.6 (3.2)	<0.001	224.5 (5.0)	208.8 (6.0)	0.051	217.5 (1.7)	209.9 (2.2)	0.008	199.9 (1.3)	196.0 (1.2)	0.034
High-density lipoprotein cholesterol (mg/dl)	48.8 (1.6)	51.2 (1.5)	0.287	44.8 (1.8)	46.3 (2.0)	0.580	48.1 (0.7)	53.5 (0.9)	<0.001	51.8 (0.7)	55.9 (0.5)	<0.001
Low-density lipoprotein cholesterol (mg/dl)	134.7 (3.0)	113.8 (2.6)	<0.001	144.8 (3.9)	130.0 (5.3)	0.028	142.2 (1.8)	129.8 (1.6)	<0.001	124.3 (1.3)	116.7 (1.1)	<0.001
Non-high-density lipoprotein cholesterol (mg/dl)	165.6 (4.6)	143.5 (3.2)	<0.001	179.7 (4.5)	162.5 (5.5)	0.019	169.4 (1.8)	156.4 (2.1)	<0.001	148.1 (1.5)	140.1 (1.2)	<0.001
Triglycerides (mg/dl)†	154.5 (9.8)	148.1 (7.4)	0.604	174.0 (10.4)	163.2 (11.9)	0.496	135.9 (4.3)	135.8 (3.7)	0.987	118.8 (3.6)	116.9 (1.7)	0.639
Triglycerides (mg/dl)‡	139.1 (9.9)	131.2 (7.1)	0.513	158.3 (9.6)	140.6 (12.1)	0.263	120.9 (3.7)	120.5 (3.0)	0.936	103.8 (3.2)	102.3 (1.6)	0.674
Apolipoprotein B (mg/dl)	113.8 (2.8)	103.2 (2.4)	0.005	121.7 (3.1)	116.9 (3.6)	0.321	115.1 (1.2)	109.6 (1.3)	0.003	101.6 (0.9)	98.7 (1.0)	0.041
**Age-adjusted**												
Total cholesterol (mg/dl)	202.5 (3.7)	185.8 (3.6)	0.002	212.9 (4.4)	198.4 (6.3)	0.070	207.4 (1.8)	202.1 (2.0)	0.048	201.6 (1.2)	198.5 (1.1)	0.062
High-density lipoprotein cholesterol (mg/dl)	46.9 (1.6)	49.7 (1.5)	0.202	42.9 (1.8)	44.6 (2.0)	0.528	46.5 (0.7)	52.2 (0.9)	<0.001	52.1 (0.7)	56.3 (0.4)	<0.001
Low-density lipoprotein cholesterol (mg/dl)	126.6 (2.9)	107.8 (2.9)	<0.001	137.0 (3.7)	122.9 (5.7)	0.046	135.3 (1.9)	124.5 (1.5)	<0.001	125.5 (1.3)	118.4 (1.1)	<0.001
Non-high-density lipoprotein cholesterol (mg/dl)	155.6 (4.5)	136.1 (3.6)	0.001	170.0 (4.1)	153.8 (5.7)	0.028	160.9 (2.0)	149.9 (2.0)	<0.001	149.5 (1.4)	142.2 (1.2)	<0.001
Triglycerides (mg/dl)†	145.2 (9.6)	141.2 (7.6)	0.743	164.9 (10.0)	155.0 (11.5)	0.518	127.9 (4.4)	129.7 (3.6)	0.755	120.2 (3.6)	118.9 (1.7)	0.750
Triglycerides (mg/dl)‡	127.6 (9.0)	123.1 (6.9)	0.683	145.6 (8.5)	130.4 (11.0)	0.283	112.3 (3.4)	113.9 (2.6)	0.713	105.1 (3.2)	104.2 (1.6)	0.797
Apolipoprotein B (mg/dl)	107.8 (2.7)	98.7 (2.6)	0.019	115.8 (2.9)	111.6 (3.8)	0.388	109.9 (1.4)	105.7 (1.2)	0.018	102.4 (0.9)	100.0 (0.9)	0.066
**Adjusted for age, gender, race or ethnicity, education, and body mass index**												
Total cholesterol (mg/dl)	201.7 (3.5)	183.7 (3.4)	<0.001	211.3 (4.7)	193.9 (6.2)	0.029	208.0 (1.9)	200.7 (2.1)	0.010	202.2 (1.2)	198.4 (1.0)	0.018
High-density lipoprotein cholesterol (mg/dl)	48.0 (1.2)	52.9 (1.4)	0.009	46.8 (2.1)	51.0 (2.5)	0.213	47.0 (0.7)	53.9 (0.8)	<0.001	51.2 (0.6)	56.3 (0.4)	<0.001
Low-density lipoprotein cholesterol (mg/dl)	125.6 (2.7)	104.8 (2.8)	<0.001	133.7 (4.3)	116.3 (5.6)	0.016	135.0 (2.0)	122.5 (1.5)	<0.001	126.2 (1.2)	118.5 (0.9)	<0.001
Non-high-density lipoprotein cholesterol (mg/dl)	153.7 (4.0)	130.8 (3.4)	<0.001	164.5 (5.3)	143.0 (5.9)	0.009	160.9 (2.0)	146.9 (2.0)	<0.001	150.9 (1.2)	142.1 (0.9)	<0.001
Triglycerides (mg/dl)†	140.6 (8.2)	129.7 (7.4)	0.324	154.1 (11.9)	134.0 (13.2)	0.258	129.6 (3.7)	124.4 (3.8)	0.319	123.4 (3.2)	118.1 (1.6)	0.141
Triglycerides (mg/dl)‡	122.9 (7.3)	111.7 (6.0)	0.224	133.2 (10.3)	109.4 (10.4)	0.107	114.3 (2.7)	109.0 (2.5)	0.148	108.0 (2.8)	103.4 (1.4)	0.146
Apolipoprotein B (mg/dl)	106.4 (2.6)	95.0 (2.5)	0.002	111.8 (3.2)	104.2 (4.0)	0.148	109.6 (1.5)	103.5 (1.2)	0.002	103.4 (0.9)	100.0 (0.7)	0.006

Participants who reported using cholesterol-lowering medications, fenofibrate, gemfibrozil, and niacin were excluded.

*P-values from linear regression analyses.

†Untransformed mean.

‡Geometric mean.

Changes in concentrations of lipids in dichotomized form are presented in Table 4. In all four groups, elevated concentrations of low-density lipoprotein cholesterol, non-high-density lipoprotein cholesterol, and apolipoprotein B decreased significantly after adjustment for age, gender, race or ethnicity, education, and body mass index. Among persons with prediabetes and normoglycemia, low concentrations of high-density lipoprotein cholesterol decreased significantly. A significant decrease in the prevalence of the lipid triad consisting of low concentrations of high-density lipoprotein cholesterol and elevated concentrations of triglycerides and apolipoprotein B was noted among participants with prediabetes and normoglycemia.

**Table 4:** Unadjusted and adjusted percentages (standard error) of lipids and apolipoprotein B among adults aged ≥20 years, by glycemic status and survey, National Health and Nutrition Examination Surveys 1988–1991 and 2005-2006

	**Diagnosed diabetes**			**Undiagnosed diabetes**			**Prediabetes**			**Normal**		
	**1988-1991**	**2005-2008**	P*	**1988-1991**	**2005-2008**	P*	**1988-1991**	**2005-2008**	P*	**1988-1991**	**2005-2008**	P*
N	207	457		92	103		566	850		2337	2539	
**Unadjusted**												
High-density lipoprotein cholesterol <40 mg/dl in men, <50 mg/dl in women	46.4 (5.8)	37.2 (3.0)	0.165	57.5 (8.7)	34.4 (6.4)	0.037	41.8 (2.8)	24.9 (1.7)	<0.001	33.3 (1.7)	22.4 (1.1)	<0.001
High-density lipoprotein cholesterol <40 mg/dl in men, <50 mg/dl in women or medication use^†^	47.9 (5.8)	40.7 (3.0)	0.275	57.5 (8.7)	37.5 (6.5)	0.071	43.4 (3.1)	26.4 (1.6)	<0.001	33.6 (1.6)	23.0 (1.0)	<0.001
Low-density lipoprotein cholesterol ≥100 mg/dl	86.1 (4.7)	42.9 (2.6)	<0.001	91.5 (3.3)	70.3 (4.9)	0.001	90.0 (1.4)	75.0 (1.7)	<0.001	74.4 (1.8)	66.0 (1.2)	<0.001
Low-density lipoprotein cholesterol ≥100 mg/dl or medication use	86.1 (4.7)	79.1 (2.4)	0.183	94.4 (2.4)	87.0 (4.9)	0.180	90.1 (1.4)	88.4 (1.3)	0.365	74.4 (1.8)	70.6 (1.2)	0.085
Non-high-density lipoprotein cholesterol ≥130 mg/dl	81.9 (5.5)	43.6 (2.8)	<0.001	91.7 (3.4)	67.5 (5.4)	<0.001	85.0 (1.5)	67.9 (1.9)	<0.001	65.2 (1.7)	57.8 (0.8)	<0.001
Triglycerides ≥150 mg/dl	44.7 (7.0)	43.9 (2.7)	0.909	54.3 (5.8)	43.0 (6.9)	0.215	33.9 (1.9)	33.2 (2.1)	0.797	24.7 (2.3)	24.7 (1.1)	0.997
Triglycerides ≥150 mg/dl or medication use^†^	46.9 (6.9)	47.8 (3.0)	0.905	54.3 (5.8)	44.1 (7.1)	0.271	35.9 (1.9)	34.6 (2.0)	0.625	24.9 (2.3)	25.1 (1.0)	0.936
Triglycerides ≥150 mg/dl or use of TG-lowering or cholesterol-lowering medications^‡^	47.6 (6.7)	73.5 (2.9)	0.001	54.3 (5.8)	67.4 (5.9)	0.121	36.9 (2.0)	50.2 (2.1)	<0.001	25.3 (2.4)	30.3 (1.3)	0.070
Apolipoprotein B ≥80 mg/dl	91.1 (3.6)	72.8 (2.1)	<0.001	95.8 (2.8)	87.9 (4.2)	0.123	92.8 (1.3)	86.6 (1.4)	0.002	81.7 (1.3)	77.2 (1.0)	0.007
Lipid triad: definition 1^Â§^	31.4 (6.5)	21.6 (2.8)	0.172	35.7 (7.7)	23.4 (5.2)	0.190	20.7 (1.7)	13.8 (1.2)	0.002	13.1 (1.5)	9.8 (0.7)	0.050
Lipid triad: definition 2^ǁ ^	33.3 (6.4)	24.8 (2.7)	0.228	35.7 (7.7)	24.0 (5.3)	0.212	23.3 (1.9)	15.2 (1.1)	0.001	13.5 (1.4)	10.4 (0.7)	0.056
Lipid triad: definition 3^Â¶^	33.3 (6.4)	26.6 (2.8)	0.342	35.7 (7.7)	25.4 (5.8)	0.287	23.8 (2.1)	16.1 (1.1)	0.002	13.7 (1.5)	10.9 (0.7)	0.102
**Age-adjusted**												
High-density lipoprotein cholesterol <40 mg/dl in men, <50 mg/dl in women	51.5 (6.8)	41.5 (3.3)	0.180	63.4 (9.5)	38.7 (7.4)	0.042	45.6 (3.0)	27.2 (1.9)	<0.001	32.4 (1.6)	21.9 (1.0)	<0.001
High-density lipoprotein cholesterol <40 mg/dl in men, <50 mg/dl in women or medication use^†^	52.1 (6.7)	44.5 (3.3)	0.293	62.2 (9.3)	41.2 (7.3)	0.078	46.6 (3.4)	28.3 (1.8)	<0.001	32.8 (1.6)	22.5 (1.0)	<0.001
Low-density lipoprotein cholesterol ≥100 mg/dl	80.9 (4.4)	40.2 (2.4)	<0.001	86.2 (3.3)	65.5 (4.6)	0.001	85.2 (1.4)	71.1 (1.7)	<0.001	75.8 (1.7)	67.1 (1.2)	<0.001
Low-density lipoprotein cholesterol ≥100 mg/dl or medication use	78.1 (4.2)	71.4 (2.2)	0.154	86.1 (2.7)	78.1 (4.3)	0.127	82.8 (1.3)	81.4 (1.4)	0.386	76.6 (1.7)	72.5 (1.2)	0.062
Non-high-density lipoprotein cholesterol ≥130 mg/dl	74.2 (5.1)	39.3 (2.6)	<0.001	83.6 (3.7)	60.5 (4.8)	<0.001	78.0 (1.6)	62.5 (1.9)	<0.001	67.1 (1.6)	59.4 (0.8)	<0.001
Triglycerides ≥150 mg/dl	38.6 (6.1)	37.7 (2.6)	0.882	47.4 (4.7)	36.6 (5.8)	0.155	29.8 (2.0)	29.3 (1.9)	0.832	25.8 (2.4)	25.7 (1.1)	0.970
Triglycerides ≥150 mg/dl or medication use^†^	40.1 (5.9)	40.6 (2.8)	0.936	46.9 (4.7)	37.2 (5.9)	0.198	31.4 (1.8)	30.3 (1.8)	0.649	26.2 (2.3)	26.3 (1.0)	0.970
Triglycerides ≥150 mg/dl or use of TG-lowering or cholesterol-lowering medications^‡^	38.1 (5.4)	58.4 (2.7)	0.001	44.2 (4.4)	52.9 (4.5)	0.160	30.3 (1.9)	41.6 (1.8)	<0.001	27.2 (2.4)	32.3 (1.3)	0.075
Apolipoprotein B ≥80 mg/dl	85.4 (3.2)	68.1 (2.1)	<0.001	90.2 (2.9)	81.9 (4.0)	0.096	87.8 (1.3)	82.0 (1.4)	0.001	83.3 (1.3)	78.5 (0.9)	0.005
Lipid triad: definition 1^Â§^	30.0 (6.4)	20.6 (2.8)	0.174	34.3 (7.2)	22.3 (5.1)	0.179	20.0 (1.8)	13.3 (1.1)	0.002	13.3 (1.5)	9.9 (0.7)	0.048
Lipid triad: definition 2^ǁ ^	30.9 (6.2)	22.9 (2.6)	0.226	33.3 (6.9)	22.0 (4.9)	0.194	21.8 (1.8)	14.2 (1.1)	<0.001	13.8 (1.4)	10.6 (0.7)	0.053
Lipid triad: definition 3^Â¶^	30.5 (6.1)	24.2 (2.7)	0.336	32.9 (6.9)	23.0 (5.3)	0.262	22.0 (2.0)	14.9 (1.1)	0.002	14.0 (1.5)	11.2 (0.7)	0.098
**Adjusted for age, gender, race or ethnicity, education, and body mass index**												
High-density lipoprotein cholesterol <40 mg/dl in men, <50 mg/dl in women	45.8 (5.7)	34.8 (3.0)	0.073	50.6 (10.4)	31.0 (7.0)	0.111	47.6 (2.9)	25.6 (1.8)	<0.001	34.0 (1.8)	21.9 (1.1)	<0.001
High-density lipoprotein cholesterol <40 mg/dl in men, <50 mg/dl in women or medication use^†^	46.7 (5.6)	37.6 (3.1)	0.131	50.3 (10.0)	33.3 (7.1)	0.157	48.6 (3.2)	26.7 (1.7)	<0.001	34.4 (1.7)	22.5 (1.0)	<0.001
Low-density lipoprotein cholesterol ≥100 mg/dl	79.8 (4.1)	38.1 (2.3)	<0.001	82.2 (3.1)	60.3 (3.8)	<0.001	85.6 (1.7)	69.2 (1.9)	<0.001	77.4 (1.8)	67.0 (1.2)	<0.001
Low-density lipoprotein cholesterol ≥100 mg/dl or medication use	77.6 (3.9)	67.8 (2.2)	0.024	82.8 (2.6)	71.8 (4.0)	0.018	83.6 (1.6)	79.3 (1.6)	0.018	78.1 (1.8)	72.4 (1.2)	0.012
Non-high-density lipoprotein cholesterol ≥130 mg/dl	72.1 (4.5)	35.6 (2.4)	<0.001	76.6 (4.0)	51.9 (3.9)	<0.001	79.2 (1.9)	59.7 (2.0)	<0.001	69.8 (1.6)	59.3 (0.8)	<0.001
Triglycerides ≥150 mg/dl	36.9 (5.3)	32.6 (2.4)	0.444	41.5 (7.2)	29.2 (6.0)	0.183	31.8 (1.9)	27.7 (1.9)	0.090	27.6 (2.4)	25.3 (1.0)	0.376
Triglycerides ≥150 mg/dl or medication use^†^	38.4 (5.1)	35.3 (2.6)	0.558	41.3 (7.1)	29.8 (6.1)	0.209	33.4 (1.6)	28.7 (1.7)	0.025	28.0 (2.4)	25.8 (0.9)	0.399
Triglycerides ≥150 mg/dl or use of TG-lowering or cholesterol-lowering medications^‡^	37.0 (4.9)	51.2 (2.8)	0.009	40.1 (5.7)	42.9 (5.2)	0.707	32.0 (1.8)	39.4 (1.8)	0.001	28.9 (2.5)	32.0 (1.2)	0.255
Apolipoprotein B ≥80 mg/dl	84.1 (3.1)	64.5 (2.0)	<0.001	85.8 (3.1)	75.5 (3.4)	0.017	88.2 (1.6)	80.0 (1.5)	<0.001	84.9 (1.2)	78.5 (0.9)	<0.001
Lipid triad: definition 1^Â§^	27.6 (5.6)	17.7 (2.5)	0.099	28.4 (8.0)	17.6 (5.0)	0.240	21.7 (1.8)	12.7 (1.1)	<0.001	14.1 (1.5)	9.8 (0.7)	0.013
Lipid triad: definition 2^ǁ ^	28.7 (5.5)	19.7 (2.4)	0.117	28.2 (7.6)	17.5 (4.9)	0.227	23.7 (1.8)	13.6 (1.0)	<0.001	14.7 (1.5)	10.4 (0.7)	0.012
Lipid triad: definition 3	28.3 (5.4)	20.8 (2.4)	0.186	27.9 (7.3)	18.1 (5.0)	0.260	23.7 (1.9)	14.1 (1.0)	<0.001	14.9 (1.5)	11.1 (0.7)	0.028

*P-values for difference of predicted marginal from log-linear regressions.

^†^Use of fenofibrate, gemfibrozil, and niacin.

^‡^Use of cholesterol-lowering medications as well as fenofibrate, gemfibrozil, and niacin.

^Â§^Definition 1 was based on measured values of concentrations of high-density lipoprotein cholesterol, triglycerides, and apolipoprotein B.

^ǁ ^Definition 2: use of fenofibrate, gemfibrozil, and niacin was counted as having elevated concentrations of triglycerides and decreased concentrations of high-density lipoprotein cholesterol.

^Â¶^Definition 3: use of cholesterol-lowering medications, fenofibrate, gemfibrozil, and niacin was counted as having elevated concentrations of triglycerides and use of fenofibrate, gemfibrozil, and niacin was counted as having decreased concentrations of high-density lipoprotein cholesterol.

Correlation coefficients among concentrations of triglycerides (log-transformed), non-high-density lipoprotein cholesterol, and apolipoprotein B are shown in Table 5. The largest correlation coefficients were noted for non-high-density lipoprotein cholesterol and apolipoprotein B and ranged from 0.90 to 0.95.

**Table 5:** Unadjusted Pearson correlation coefficients among triglycerides, non-high-density lipoprotein cholesterol, and apolipoprotein B among adults aged > =20 years, National Health and Nutrition Examination Survey 2005-2008

**Glycemic status**	**Ln(TG) and non-HDLC**	**Ln(TG) and apo B**	**non-HDLC and apo B**
Diagnosed diabetes	0.40	0.40	0.96
Undiagnosed diabetes	0.45	0.37	0.95
Prediabetes	0.51	0.45	0.90
Normal	0.51	0.49	0.95

apo B = apolipoprotein B; non-HDLC = non-high-density lipoprotein cholesterol; TG = triglycerides.

## Discussion and conclusion

Several key points emerged from our analyses. First, the lipid profile of adults with undiagnosed diabetes during 1988–1991 was the worst of the four groups; these participants had the lowest adjusted mean concentration of high-density lipoprotein cholesterol or the highest adjusted mean concentrations of total cholesterol, non-high-density lipoprotein cholesterol, triglycerides, and apolipoprotein B. By 2005–2008, however, this group no longer uniformly had the worst mean concentrations of lipids and apolipoprotein B. Second, unadjusted and adjusted mean concentrations of several lipids improved from 1988–1991 to 2005–2008 among adults in all four groups. Third, some of this improvement was likely a consequence of the increased use of cholesterol-lowering medications and, to a lesser degree, the increased use of triglyceride-lowering medications. Fourth, the age-adjusted prevalence of the atherogenic lipid triad, as we defined it, decreased significantly only among adults with prediabetes and normoglycemia.

Adults with diabetes experienced the largest absolute decreases in mean concentrations of total cholesterol, low-density lipoprotein cholesterol, non-high-density lipoprotein cholesterol, and apolipoprotein B. All of these lipids have been shown to be directly related to the risk for cardiovascular disease, although a long-running debate about the superiority of one lipid parameter over another in predicting risk of cardiovascular disease remains unresolved [15-19]. Thus, the improvement in all of these lipid parameters is encouraging and suggests that the risk for cardiovascular disease, at least from lipids, has declined over time. Previous studies have shown that morbidity and mortality from cardiovascular disease among adults with diabetes have declined in the United States [20,21].

The rapidly increasing use of cholesterol-lowering medications is undoubtedly a major contributor to the improvements in these lipids. The increased use of such medications among adults with diabetes has been reported previously [9]. The contribution of other factors is less clear. The intake of dietary fat in the United States has not changed appreciably in recent decades [22], and, thus, it seems unlikely that changes in the intake of this macronutrient provide a ready explanation for the observed reduction in mean concentrations of these lipids. One factor that should have worked against favorable improvements in lipid concentrations is the strong increase in body mass index; increased body mass index generally correlates with increased concentrations of low-density lipoprotein cholesterol, non-high-density lipoprotein cholesterol, apolipoprotein B, and triglycerides as well as decreased concentrations of high-density lipoprotein cholesterol. The fact that improvements in concentrations of various lipids were observed in the face of the rising prevalence of obesity testifies to the presence of other powerful secular trends that were able to offset the potentially deleterious impact of obesity on lipid concentrations.

In contrast, the mean concentrations of triglycerides did not change significantly among patients with diabetes. The mean concentration of high-density lipoprotein cholesterol increased significantly only when covariates including body mass index were taken into account. Although the use of medications known to influence concentrations of these lipids increased significantly, especially among adults with diagnosed diabetes, the use of these agents is far less common than that of cholesterol-lowering medications. Hence, the use of fenofibrate, gemfibrozil, and niacin is unlikely to have substantially influenced the observed mean concentrations. Dietary practices can affect concentrations of triglycerides. However, the intake of fat has changed little since 1999–2000 whereas the intake of carbohydrates has decreased [22]. Concentrations of high-density lipoprotein cholesterol are influenced by physical activity, alcohol use, and other factors. Trends in physical activity in the United States remain clouded because of inconsistency in recording physical activity in national surveys. Data from the National Health Interview Survey from 1999 to 2010 shows that some improvement in the percentage of adults meeting current guidelines has taken place [23]. A recent analysis of energy expenditure in the workplace concluded that a reduction in energy expenditure has occurred over five decades [24]. Per capita alcohol consumption has increased slightly from a little over 2.1 gallons during 1997–1998 to about 2.3 gallons during 2009 [25].

Among people with diabetes, changes in glycemic control can also affect lipid parameters. For example, in a study of 73 patients with type 2 diabetes, improved glycemic control was associated with favorable changes in high-density lipoprotein cholesterol, apolipoprotein A1 and the ratio of apolipoprotein A1 to apolipoprotein B [26]. Because data indicate that glycemic control has improved in the United States [10,27], this development may have contributed to improving the lipid profiles of people with diabetes.

An interesting observation from our analyses is that adults with undiagnosed diabetes and prediabetes showed evidence of having worse lipid profiles than adults with diagnosed diabetes. The high uptake of cholesterol-lowering medications by adults with diagnosed diabetes likely improved the lipid profile of these adults in comparison with the lipid profiles of adults with undiagnosed diabetes and prediabetes. Nevertheless, mean concentrations of lipids generally changed in a favorable direction among adults with undiagnosed diabetes and prediabetes, although the magnitude of the change was generally smaller than that achieved by adults with diagnosed diabetes. This observation suggests that adults with undiagnosed diabetes and prediabetes are at increased cardiovascular risk from uncontrolled lipid abnormalities and that, once diabetes is diagnosed, adults with previously undiagnosed diabetes will benefit from treatment to manage dyslipidemias.

Apolipoprotein B is the protein that constitutes the principal structural element of lipoprotein particles for very-low-density lipoprotein (VLDL), intermediate-density lipoprotein (IDL), and LDL. Two forms of apolipoprotein B have been identified: apolipoprotein B48 and apolipoprotein B100. The latter is generally measured in assays of apolipoprotein B such as the present study. Apolipoprotein B100 is produced in the liver, incorporated into VLDL particles, and subsequently secreted into the circulation where the particle is transformed into LDL. Because there is one molecule of apolipoprotein B per lipoprotein particle, concentrations of apolipoprotein B reflect the number of VLDL, IDL, and LDL particles. Apolipoprotein B is recognized by cell receptors, leading to the uptake of the lipoprotein particle. Numerous studies have shown that concentrations of apolipoprotein B are directly related to cardiovascular risk, and some have argued that apolipoprotein B is a better lipid measure for assessing cardiovascular risk than are other lipid parameters such as low-density lipoprotein cholesterol and non-high-density lipoprotein cholesterol [15,17,18] although considerable disagreement persists [16,19]. Because apolipoprotein B is not routinely measured in clinical practice at present, alternative methods to derive estimates of concentrations of apolipoprotein B have been pursued [28].

In persons with diabetes, a limited number of studies has shown that concentrations of apolipoprotein B predict cardiovascular morbidity and mortality [29-32], but not always better than other lipid markers [32]. Concentrations of apolipoprotein B have also been shown to be associated with diabetic retinopathy [33], microalbumiuria [34,35], carotid atherosclerosis [36,37], and coronary artery calcification [38]. Thus, the drop in mean concentration of apolipoprotein B in adults with diabetes in the present study, which was consistent with the decreases in mean concentrations of low-density lipoprotein cholesterol and non-high-density lipoprotein cholesterol, suggests a lessening of cardiovascular risk among people with diabetes. The data suggest that the use of lipid-lowering medications accounted for a substantial portion of the decrease.

Current guidelines from the American Diabetes Association and the American College of Cardiology Foundation call for reducing concentrations of apolipoprotein B to less than 80 mg/dl in people who have diabetes in addition to at least one additional major risk factor for cardiovascular disease [39]. Elevated concentrations of apolipoprotein B were the most pronounced dyslipidemia among all four groups of adults. Pharmacologic approaches to accomplishing this goal in people with diabetes rely mostly on the use of statins [40]. Insulin treatment in patients with type 2 diabetes has been shown to lower concentrations of apolipoprotein B [41]. Potential nonpharmacological approaches includes increased physical activity and dietary change.

The findings of the present study are subject to several limitations. First, methods and laboratories used to conduct the lipid measurements changed during the study period. Although strict quality control procedures were implemented in all surveys, direct comparisons were not done. Supportive of the validity of our observations, however, is that the changes in all the atherogenic indices- low-density lipoprotein cholesterol, non-high-density lipoprotein cholesterol, and apolipoprotein B- were similar. Second, the sample sizes for some of the groups, particularly diagnosed diabetes and undiagnosed diabetes, were small resulting in limited statistical power to detect significant changes in lipid concentrations. The available sample sizes also precluded us from performing stratified analyses by various sociodemographic or other variables.

In conclusion, mean concentrations of several lipids and apolipoprotein B decreased significantly from 1988–1991 to 2005–2008 among adults with diagnosed diabetes. However, no statistically significant changes in concentrations of triglycerides transpired. The rapid increase in the use of lipid-lowering medications, especially among adults with diagnosed diabetes, likely was a factor in the observed changes. Despite these improvements, sizeable percentages of adults with diabetes continue to have elevated concentrations of low-density lipoprotein cholesterol, triglycerides, and especially apolipoprotein B. Thus, further progress in adequately managing dyslipidemias in adults with diabetes, and thereby lessening their risk for cardiovascular morbidity and mortality, remains to be achieved.

## Abbreviations

NHANES: National Health and Nutrition Examination Survey.

## Competing interests

The authors declare no competing interests.

## Authors’ contributions

ESF conceived of the study, researched data, and wrote the manuscript. CL consulted in the analysis of the data and helped write the manuscript. AS participated in the design of the study and helped write the manuscript. All authors read and approved the final manuscript.

## Disclaimer

The findings and conclusions in this article are those of the authors and do not represent the official position of the Centers for Disease Control and Prevention.
